# Once Upon a Time Without DMF: Greener Paths in Peptide and Organic Synthesis

**DOI:** 10.3390/molecules31030536

**Published:** 2026-02-03

**Authors:** Antonia Scognamiglio, Elisa Magli, Giuseppe Caliendo, Elisa Perissutti, Vincenzo Santagada, Beatrice Severino

**Affiliations:** 1 Department of Pharmacy, School of Medicine, University of Naples Federico II, Via D. Montesano, 49, 80131 Napoli, Italy; antonia.scognamiglio@unina.it (A.S.); caliendo@unina.it (G.C.); elisa.perissutti@unina.it (E.P.); santagad@unina.it (V.S.); 2 Department of Public Health, School of Medicine, University of Naples Federico II, Via Pansini, 5, 80131 Napoli, Italy; elisa.magli@unina.it

**Keywords:** DMF alternatives, sustainable solvents, peptide synthesis, organic synthesis, green chemistry, toxicity, ECHA restrictions

## Abstract

N,N-Dimethylformamide (DMF) has been a cornerstone solvent in both peptide and organic synthesis due to its excellent solubilizing properties and chemical stability. However, its use has raised significant health and environmental concerns. DMF is classified as a substance of very high concern (SVHC) by the European Chemicals Agency (ECHA) due to its reproductive toxicity and potential for skin absorption, leading to liver damage upon prolonged exposure. Consequently, restrictions on its use have been introduced, encouraging the scientific community to seek safer, more sustainable alternatives. This review provides a comprehensive analysis of the existing literature on alternative solvents to DMF, identifying current gaps or problems, and offering recommendations for future research.

## 1. Introduction

N,N-Dimethylformamide (DMF) is a colorless, highly polar, aprotic solvent. It is miscible with water and most organic solvents, making it an ideal medium for a wide range of chemical reactions. The chemico-physical characteristics—such as melting and boiling range, density, viscosity, surface tension, partition coefficient, flash point, autoignition temperature, and electrical conductivity—of DMF and related dipolar aprotic solvents, such as N-methyl-2-pyrrolidone (NMP), and N,N-dimethylacetamide (DMAc) fall within the typical values observed for common organic solvents. What makes them uniquely valuable in the chemical industry is their remarkable polarity.

Dipolar aprotic solvents are recognized for their high dielectric constant and for being non-protic, properties that strongly influence solvation behavior. The solvent’s high polarity and dipolar moment (approximately 3.86 D) enable it to dissolve both polar and nonpolar compounds effectively. This characteristic is particularly valuable in peptide synthesis, where DMF facilitates the solubilization of amino acids and coupling reagents, as well as resin swelling, thereby promoting efficient reactions. Additionally, DMF’s ability to stabilize reactive intermediates and transition states contributes to its widespread use in organic synthesis. In solid-phase peptide synthesis (SPPS), DMF serves multiple roles: it swells the resin, allowing for better diffusion of reagents; it dissolves amino acids and coupling reagents; and it does not react with the peptide chain, ensuring the integrity of the growing sequence. Moreover, DMF is utilized in various organic reactions, including the Vilsmeier–Haack formylation and Bouveault aldehyde synthesis, due to its ability to form reactive intermediates that facilitate these transformations. Despite its advantages, the use of DMF has raised significant health and environmental concerns. It is associated with significant human health risks, including hepatotoxicity, reproductive toxicity, and skin absorption, which have led regulatory agencies to classify it as a substance of very high concern (SVHC) under the European Chemicals Agency (ECHA) framework.

Consequently, restrictions on its use have been introduced, encouraging the scientific community to seek safer, more sustainable alternatives in accordance with green chemistry principles.

Accordingly, this review first addresses the toxicity and regulatory concerns associated with DMF and related dipolar aprotic solvents, then surveys the most promising sustainable alternatives, describing their key physicochemical properties and preparation methods, and finally highlights representative applications of these solvents as drop-in replacements for DMF in organic and peptide synthesis. Selected examples include Pd-catalyzed cross-coupling reactions, amide bond formation, solid- and solution-phase peptide synthesis, as well as microwave- and ultrasound-assisted methodologies, providing practical insight into currently available and viable solvent substitution strategies.

## 2. Toxicity and Regulatory Concerns of DMF and Related Solvents

Polar aprotic solvents such as DMF, NMP, DMAc, and N-ethyl-2-pyrrolidone (NEP) are widely employed in peptide and organic synthesis but have been classified under Article 57(c) of the REACH Regulation (EC 1907/2006) as substances toxic for reproduction and consequently designated as Substances of Very High Concern (SVHCs) between 2011 and 2012 [1]. Once included in the Candidate List for Authorization, these solvents became subject to detailed risk management evaluation within the EU/EEA framework (covering also Norway, Iceland, and Liechtenstein). The REACH process, which combines registration, evaluation, and subsequent risk management, gradually shifted from the mere classification of substances to more stringent measures such as Authorization or Restriction, in order to stimulate the substitution of hazardous chemicals [2].

According to the harmonized classification and labeling (CLP00) approved by the European Union, DMF may damage the unborn child, is harmful in contact with skin, causes serious eye irritation, and is harmful if inhaled. Additionally, the classification provided by companies to ECHA in REACH registrations identifies that this substance may damage fertility or the unborn child and is a flammable liquid and vapor.

For this solvent, inclusion as an SVHC occurred in December 2012, followed by a proposal for Authorization in 2014. In 2016–2018, regulators considered grouping DMF with NMP and DMAc, given their shared toxicological profiles [3]. This process culminated in a restriction proposal for DMF in October 2018, with public consultation and socio-economic analysis finalized by 2019 [4]. Initially, DNEL values for occupational exposure were set at 3.2 mg/m^3^ (inhalation) and 0.79 mg/kg/day (dermal) but were later revised to 6 mg/m^3^ and 1.1 mg/kg/day, respectively [4]. The restriction was formally adopted through Commission Regulation (EU) 2021/2030, and from 12 December 2023, DMF cannot be manufactured, marketed, or used above 0.3% *w*/*w* in mixtures unless employers can demonstrate compliance with the updated exposure limits and risk management conditions [5,6].

A comparable regulatory pathway applied to NMP, which since May 2020 cannot be placed on the market in concentrations ≥ 0.3% without full inclusion of chemical safety assessments; the derived limits were established at 14.4 mg/m^3^ (inhalation) and 4.8 mg/kg/day (dermal) [7].

DMAc entered the SVHC Candidate List in 2011 and was grouped with DMF and NMP in 2018; a restriction proposal was submitted in 2019, followed by a call for evidence in early 2020 [8]. The current regulatory approach, therefore, does not entail an outright ban but rather the imposition of stricter occupational exposure thresholds, reflecting the fact that viable substitutes are often structurally similar reprotoxic amides.

NEP is registered under the REACH Regulation from 2019, and it is manufactured in and/or imported to the European Economic Area, at ≥100 to <1000 tons per annum. This substance is used by consumers, by professional workers (widespread uses), in formulation or re-packing, at industrial sites, and in manufacturing. Regulation (EU) 2025/1090 amended Annex XVII to REACH by imposing binding and detailed restrictions on the use and placing on the market of DMAc and NEP. The Authority’s assessment was based on the systemic effects of these substances and on the DNEL values calculated for different routes of exposure, both short-term and long-term. The new restrictions (N. 80 and 81) therefore stipulate that from 23 December 2026, DMAc and NEP may not be placed on the market in concentrations ≥ 0.3%, used in industrial or professional settings, and used for the production of synthetic or artificial fibers with DMAc (in this case, a transitional period extended to 23 June 2029 is provided for). Then, the new DNEL values for DMA are 13 mg/m^3^ (inhalation) and 1.8 mg/kg/day (dermal), while for NEP, 4.0 mg/m^3^ (inhalation) and 2.4 mg/kg/day (dermal) [9].

It is expected that, in line with DMF, NMP, DMAc, NEP, and related solvents will eventually be subject to analogous restrictions within the EU, whereas such measures are not currently foreseen in the UK or under the US TSCA framework [6,8].

## 3. Sustainable Alternatives to DMF

**Figure 1:**
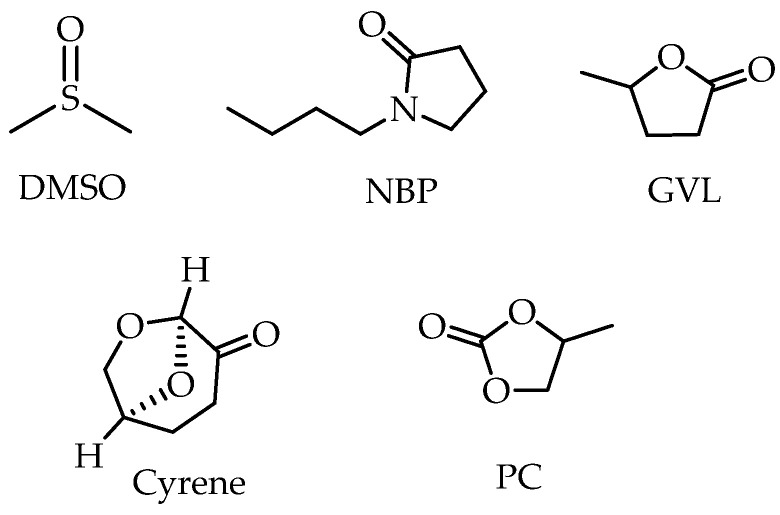
Candidate solvents proposed as environmentally friendly and low-hazard options to DMF and related solvents.

To address the limitations of DMF, several alternative solvents have been proposed. These alternatives aim to provide similar or superior solubilizing properties while minimizing health and environmental risks. Some of the proposed alternatives are depicted in Figure 1.

For each solvent, a Solvent Identity Card was developed, providing a concise overview of its physicochemical properties, safety and environmental profile, and green chemistry aspects. Physicochemical properties were extracted from the ECHA databases.

### 3.1. Dimethyl Sulfoxide (DMSO)

DMSO is a polar aprotic solvent with high solubilizing power, making it a suitable alternative to DMF in many reactions. It has been successfully employed in peptide synthesis and organic reactions, showing performance that, in some cases, approaches that of DMF (e.g., comparisons among DMF, NBP, and DMSO in SPPS) [10]. However, DMSO’s ability to penetrate biological membranes and affect membrane integrity has been demonstrated via both simulation and experimental studies, which reveal increases in membrane permeability, pore formation, and cytotoxic effects in mammalian cells at relatively low concentrations [11,12].

#### Preparation

**Figure 2:**
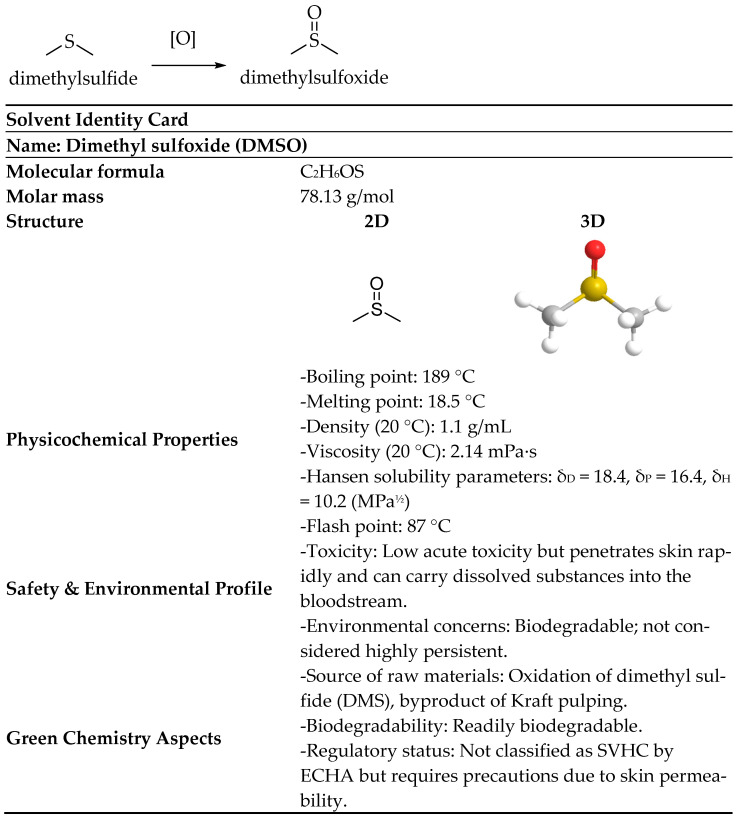
Preparation of DMSO.

Dimethyl sulfoxide (DMSO) is not a naturally abundant compound; its commercial availability relies on industrial synthesis, primarily from dimethyl sulfide (CH_3_–S–CH_3_, DMS), obtained during the Kraft process for wood pulping (a major source of lignin byproducts). Vanadium pentoxide (V_2_O_5_), molybdenum oxides, or other heterogeneous catalysts are often used to achieve selective oxidation to the sulfoxide stage, avoiding over-oxidation to dimethyl sulfone (DMSO_2_) (Figure 2) [13,14].

### 3.2. N-Butylpyrrolidinone (NBP)

NBP is a green solvent with low toxicity and excellent solvating properties. It has been utilized in solid-phase peptide synthesis (SPPS), offering advantages such as reduced side reactions and improved product yields compared to DMF. For example, it has been shown that using NBP instead of DMF resulted in lower or equal racemization for amino acids prone to this side reaction and a marked reduction in aspartimide formation compared to DMF [15]. The dipolarity (π*) of NBP is slightly lower than would be expected for a full substitution of traditional dipolar aprotic solvents. Extending the N-alkyl chain from NMP to NBP reduces solvent dipolarity, yet the increased electron-donating character of the alkyl group enhances its hydrogen-bond accepting strength. As with all dipolar aprotic media, these solvents lack hydrogen-bond donor ability (α = 0) but retain relatively high Reichardt polarity values [16].

#### Preparation

**Figure 3:**
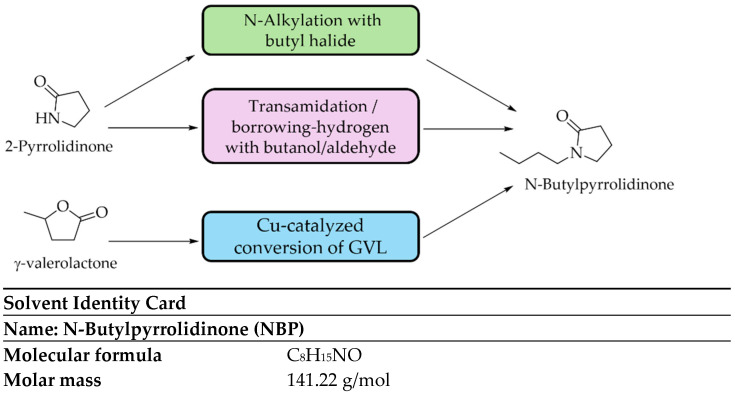

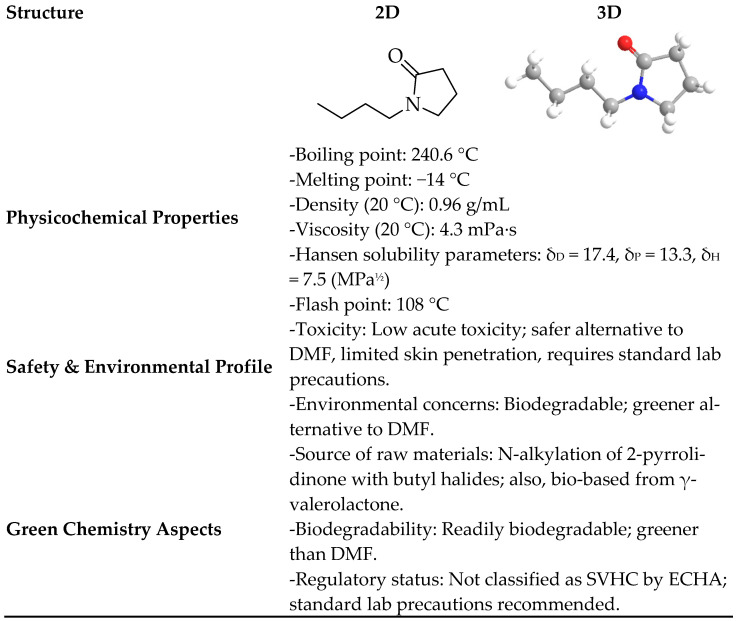
Preparation of NBP.

NBP can be synthesized through several complementary approaches, each with specific advantages and limitations in terms of sustainability and scalability (Figure 3). The most classical route relies on the N-alkylation of 2-pyrrolidinone with butyl electrophiles (such as 1-bromobutane, 1-chlorobutane, or butyl tosylate) under basic conditions, often using carbonate or alkoxide bases. After reaction, purification by distillation or aqueous washes is necessary to remove unreacted halides and residual base, yielding material of sufficient purity for peptide synthesis applications [16,17].

Alternative halide-free pathways include transamidation of substituted pyrrolidones or borrowing-hydrogen/reductive amination strategies in which 2-pyrrolidinone reacts with alcohols or aldehydes under hydrogen transfer conditions in the presence of suitable catalysts. These methodologies avoid halogenated byproducts and are considered more environmentally friendly, although further optimization is required to suppress side reactions and enable cost-effective industrial scale-up [18]. More recently, significant progress has been achieved in bio-based synthesis routes. In particular, γ-valerolactone (GVL), a renewable platform molecule obtained from lignocellulosic biomass, can be directly converted into N-alkylpyrrolidinones, including NBP. Using heterogeneous copper catalysts with highly dispersed nanoparticles, Cavuoto and co-workers reported the efficient conversion of GVL into N-butylpyrrolidinone under relatively mild conditions, thereby minimizing the need for noble metals or harsh reaction environments [19]. This approach provides an attractive example of integrating renewable feedstocks and sustainable catalysis for the scalable production of greener solvents.

### 3.3. γ-Valerolactone (GVL)

GVL is a bio-based solvent obtained from renewable resources and has recently attracted attention as a sustainable alternative to DMF in synthetic chemistry. Its use has been demonstrated in the preparation of zeolitic imidazolate frameworks such as ZIF-90, where it delivered high product yields under mild, room-temperature conditions [20]. Beyond porous materials, GVL has been exploited in organic transformations due to a dielectric constant similar to that of the widely employed reaction media. GVL’s high boiling point (207 °C) is coupled with remarkable stability under unconventional energy inputs—such as microwave irradiation and electrochemistry—often displaying efficiencies comparable to those of classical polar aprotic solvents [21]. Owing to its compatibility with both water and oxygen, GVL is particularly well suited for low-waste synthetic methodologies aimed at producing organic compounds, while consistently limiting metal catalyst leaching. Also, toxicological and environmental assessments confirm its low acute toxicity in aquatic organisms and mammalian cell models and its ready biodegradability, endorsing its potential as a safer, greener alternative [22].

#### Preparation

**Figure 4:**
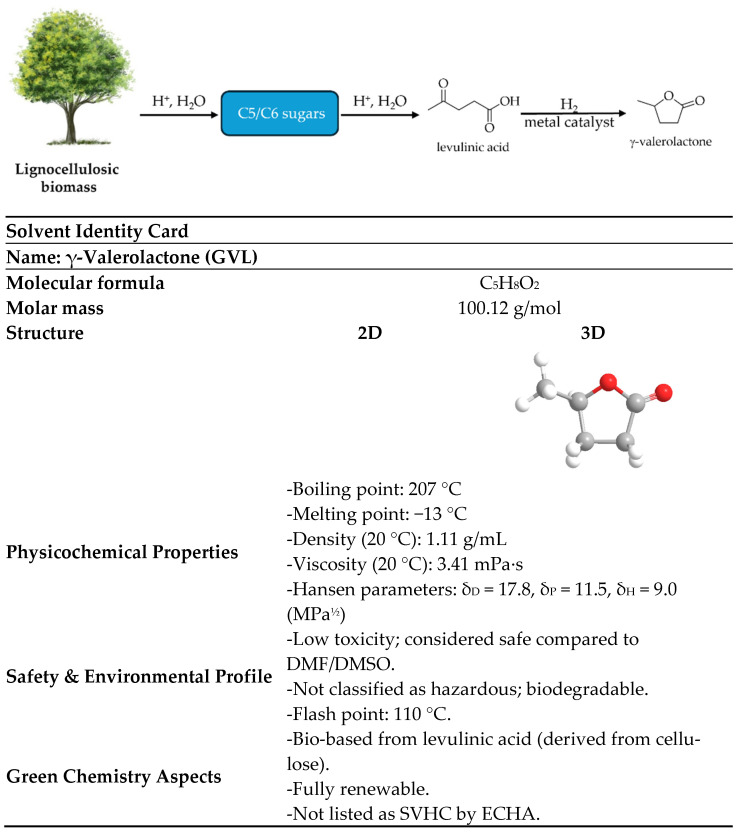
Preparation of GVL.

GVL is synthesized from biomass-derived precursors (Figure 4). The general pathway is as follows: the formation of levulinic acid from carbohydrates or lignocellulosic biomass via acid catalysis and dehydration; the hydrogenation of levulinic acid using supported metal catalysts (e.g., Pd-Cu on ZrO_2_) in water or other benign media to afford GVL [23]. The process is often designed to be cascade (multi-product) so that side streams are valorized (e.g., producing 1,4-pentanediol, 2-MeTHF in addition to GVL under certain conditions). Purification typically involves separation from the aqueous phase, distillation, and removal of by-products [23,24].

### 3.4. Cyrene^TM^ (Dihydrolevoglucosenone)

Cyrene^TM^ is a novel, bio-based solvent that has shown promise as a sustainable alternative to DMF. Kamlet–Taft and Hansen solubility parameter analyses place Cyrene in a solvent space comparable to that of NMP. Notably, Cyrene undergoes a reversible equilibrium between its ketone and geminal diol forms, with solubility enhancements correlated to this amphiphilic species, supporting its classification as a solvent with tunable solvation behavior. It has been successfully used in organic synthesis, offering comparable performance to traditional solvents while being less toxic and more environmentally friendly.

#### Preparation

**Figure 5:**
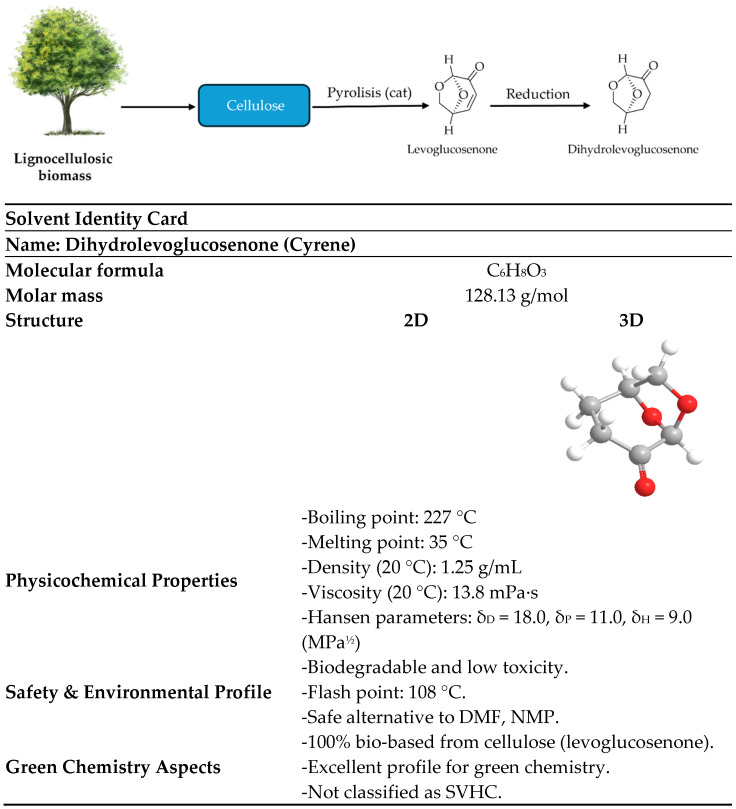
Preparation of Cyrene.

Cyrene is produced from renewable cellulose feedstocks via a two-step route (Figure 5). Firstly, levoglucosenone (LGO) is generated from cellulosic biomass by thermochemical conversion processes (e.g., pyrolysis or acid-catalyzed dehydration), followed by isolation. Subsequently, LGO undergoes selective hydrogenation under controlled conditions using heterogeneous catalysts to yield dihydrolevoglucosenone (Cyrene). The hydrogenation step must avoid over-reduction or side reactions such as dimerization or aldol condensation of Cyrene molecules under basic and acidic conditions [25,26]. Purification often involves removal of residual catalysts, distillation, or extraction steps to achieve a high-purity solvent suitable for synthetic applications.

### 3.5. Propylene Carbonate (PC)

PC is a cyclic carbonate solvent derived from propylene oxide. It is a clear, odorless liquid with high polarity (dielectric constant ≈ 64 at 25 °C), low vapor pressure, and excellent chemical stability, making it an attractive green alternative to DMF in several synthetic applications [27]. PC is miscible with water and many organic solvents, while being biodegradable and having relatively low toxicity compared to traditional dipolar aprotic solvents. PC is comparable to acetone and acetonitrile, common solvents used in organic synthesis, in terms of polarity (δP) and hydrogen-bond accepting properties (δH); these characteristics gave an approximate indication of comparable solvating ability [28]. Serving the role traditionally fulfilled by DMF, PC finds application in metal-catalyzed cross-coupling, SN_2_ chemistry, and peptide synthesis. Although less commonly employed than DMF or NBP, PC has shown promise due to its ability to dissolve amino acids and coupling reagents efficiently, while reducing hazardous exposure risks. Its high boiling point (242 °C) also makes it compatible with microwave-assisted synthesis, enabling rapid reaction rates under controlled conditions [29,30].

#### Preparation

**Figure 6:**
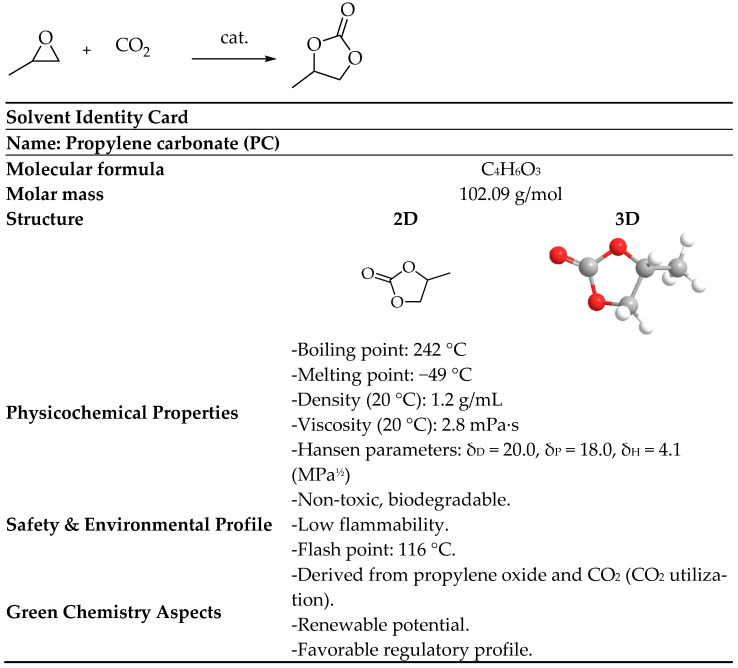
Preparation of PC.

Propylene carbonate is mainly synthesized by fixation of carbon dioxide into epoxides, representing one of the most efficient and sustainable uses of CO_2_ as a C1 feedstock (Figure 6) [31]. The most common industrial route involves the reaction of propylene oxide with CO_2_ in the presence of heterogeneous catalysts such as quaternary ammonium salts, ionic liquids, or metal complexes. This reaction is 100% atom-economic and represents a benchmark in green chemistry. Alternative transesterification methods, in which urea or ethylene carbonate reacts with propylene glycol to yield propylene carbonate, have also been reported, though this route is less common compared to direct CO_2_ fixation [32].

## 4. Applications of DMF Alternatives in Peptide and Organic Synthesis

### 4.1. Solution-Phase Synthesis

**Scheme 1:**
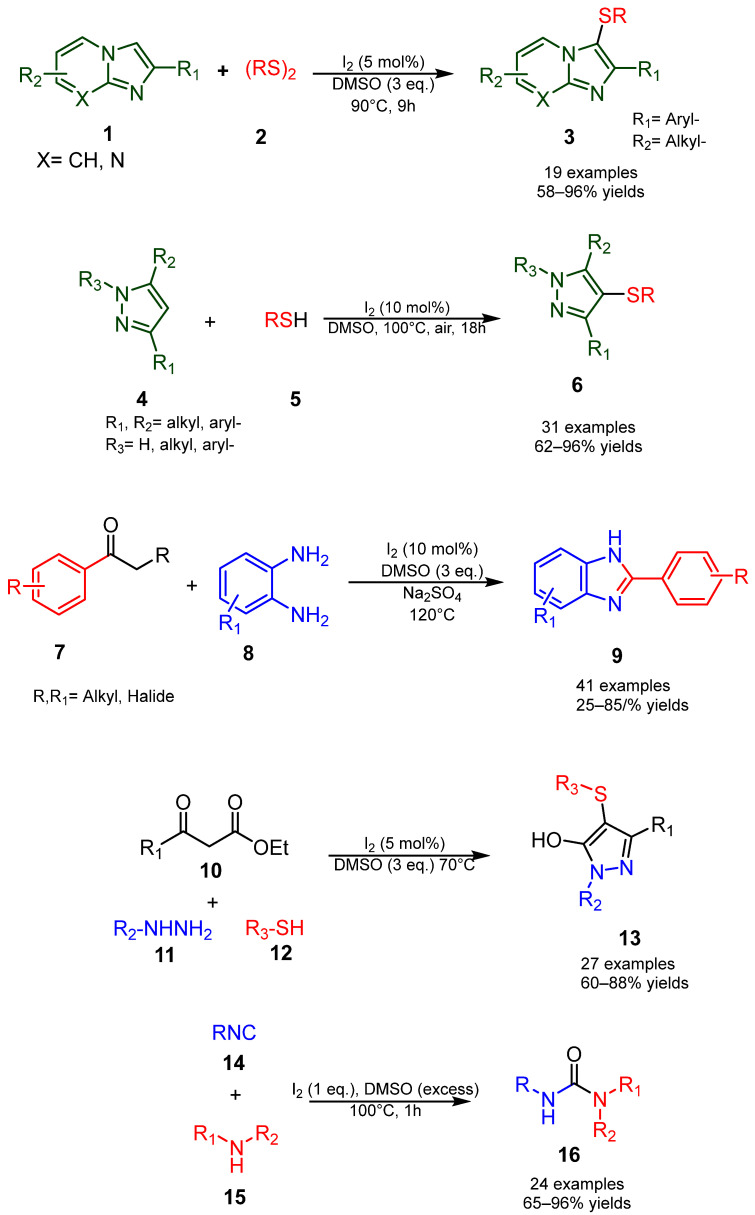
Formation of different types of C-X bonds catalyzed by I_2_/DMSO.

Alternative solvents have been successfully employed in classical solution-phase organic synthesis. For instance, DMSO has been used as a replacement for DMF in amide bond formation and nucleophilic substitution reactions, where its high polarity and ability to stabilize transition states have proven advantageous [33]. More recent studies have further highlighted its role in stabilizing transition states and weak intermolecular interactions in amide-containing [34], as well as in promoting nucleophilic substitution processes under transition-metal-free conditions [35]. Moreover, the use of DMSO in catalytic systems such as I_2_/DMSO has been demonstrated to efficiently facilitate C–heteroatom bond formation, as disclosed in the synthesis of some heterocycles and sulfur compounds, such as chalcogenated imidazo[1,2-a]pyridines (**3**) sulfonated pyrazoles (**10**), as well as benzoimidazoles (**6**), asymmetric ureas (**13**) and oxazoles (**16**), underscoring its versatility beyond traditional polar aprotic solvents (Scheme 1) [36].

**Scheme 2:**
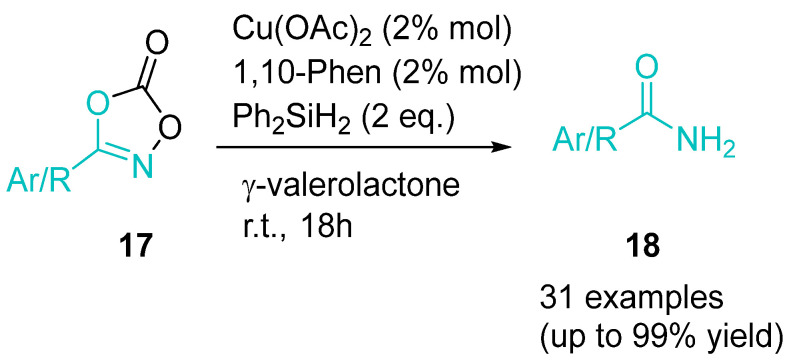
Synthesis of primary amides through reductive N–O cleavage of dioxazolones.

Similarly, γ-valerolactone (GVL) has emerged as one of the most promising bio-based solvents for solution-phase organic synthesis. A representative example is its use in amide bond formation; GVL was shown to efficiently replace DMF in carbodiimide-mediated couplings, providing comparable yields while significantly reducing the environmental burden [37]. More recently, in a Cu(OAc)_2_/phenanthroline-catalyzed reductive N–O bond cleavage of dioxazolones (**17**), GVL proved superior to DMF, affording high yields of primary amides (**18**) under mild conditions and room temperature (Scheme 2) [38].

**Scheme 3:**
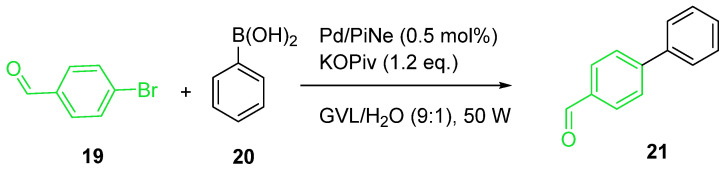
Suzuki–Miyaura cross-coupling in GVL catalyzed by Pd/PiNe, a biowaste-derived heterogeneous catalyst.

**Scheme 4:**
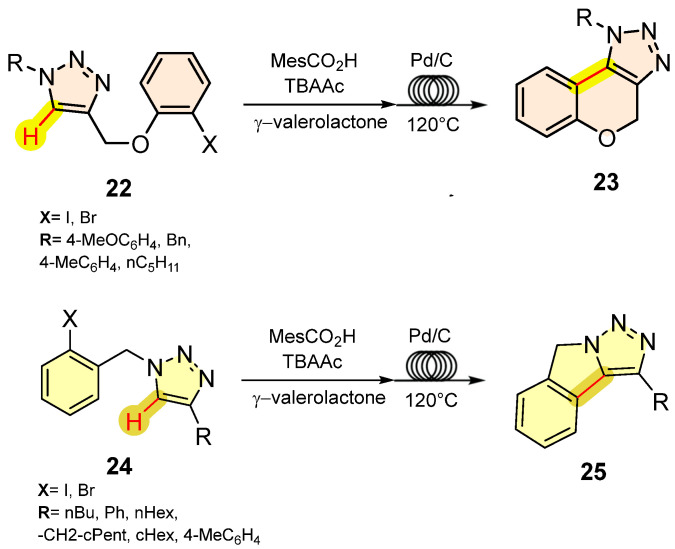
Synthesis of chromeno-fused and isoindoline-fused triazoles via C–H functionalization of 1,2,3-triazoles in γ-valerolactone.

GVL has also been explored as a green alternative in transition-metal-catalyzed transformations. For instance, it was successfully applied in Suzuki–Miyaura cross-coupling between 4-bromobenzaldehyde (**19**) and phenylboronic acid (**20**) as model substrates. Here, its use not only supported efficient C–C bond formation but also contributed to lower E-factors and improved sustainability metrics compared to DMF-based protocols (Scheme 3) [39]. The potential use of this solvent within a recovery and reuse system has been explored by Vaccaro and co-workers, who reported a regioselective C–H functionalization of 1,2,3-triazoles carried out in γ-valerolactone (GVL) under continuous-flow conditions. The process afforded cyclized products, chromeno-fused triazoles (**23**) and isoindoline-fused triazoles (**25**), in excellent yields (79–91%) across a series of substrates, while also demonstrating the feasibility of solvent recovery and direct reuse without additional purification steps (Scheme 4) [40].

In addition to their advantages, a significant drawback of high-boiling alternative solvents, such as DMSO (b.p. 189 °C) and GVL (b.p. 207 °C), is their difficult removal from reaction mixtures. This characteristic may complicate product isolation and purification compared to DMF (b.p. 153 °C). For example, in solution-phase amide bond formation in DMSO, an additional liquid–liquid extraction step using water-saturated organic solvents (e.g., ethyl acetate or diethyl ether) is often required to separate the reaction product efficiently [41]. In the case of GVL, its high boiling point and partial miscibility with water frequently necessitate adsorption on silica or liquid–liquid partitioning with brine prior to chromatographic purification [42]. Despite these challenges, strategies such as in situ product precipitation, use of biphasic solvent systems, or selective crystallization have been employed to overcome removal difficulties, thus improving the overall sustainability of the processes [43].

**Scheme 5:**
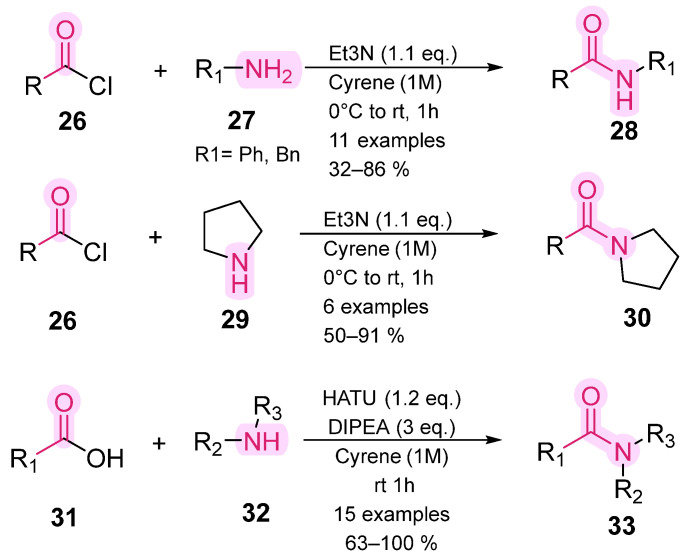
Amide bond formation in Cyrene.

**Figure 7:**
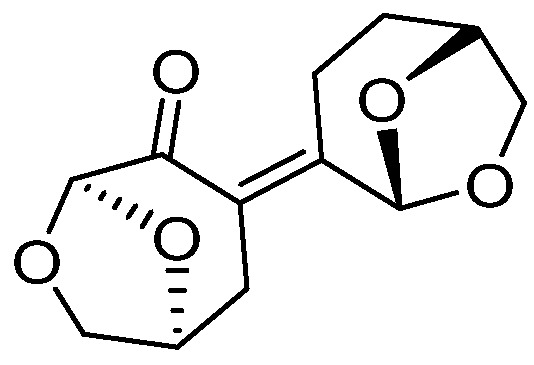
Dimer product of Cyrene’s self-aldol condensation.

In recent years, Cyrene has been proposed as an environmentally benign solvent for amide bond formation, palladium-catalyzed cross-coupling reactions, and urea synthesis. This bio-based solvent facilitates amide synthesis by means of acid chlorides (**26**) and amines (**27**, **29**) in the presence of triethylamine or carboxylic acid (**31**) and widely applied coupling agents (Scheme 5) [44,45]. However, the use of Cyrene with a base must be adequately considered, as many inorganic bases react with Cyrene, promoting the aldol addition and self-condensation to form dimers (Figure 7), which are prone to further polymerization. However, the reaction with amines occurs upon heating the reaction mixture, and the use of triethylamine at room temperature is well tolerated.

**Scheme 6:**
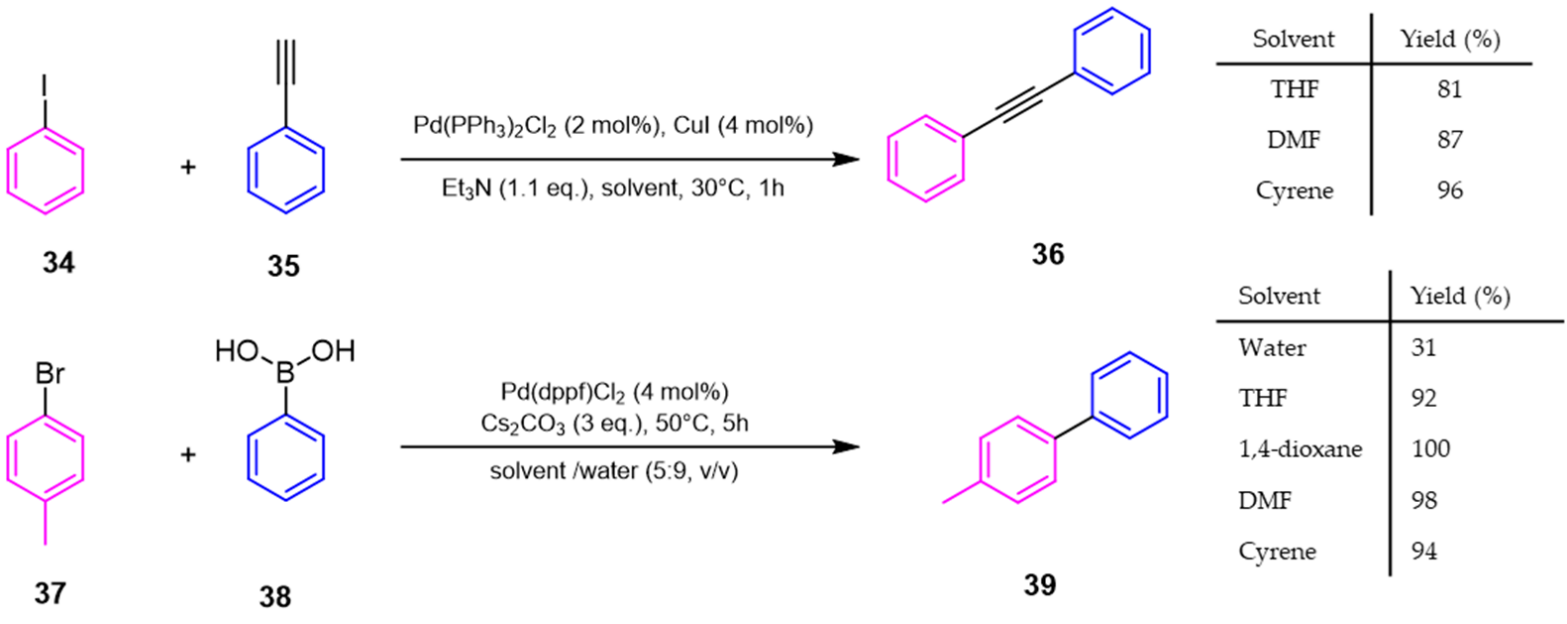
Solvent effect on the Suzuki–Miyaura and Sonogashira coupling.

**Scheme 7:**
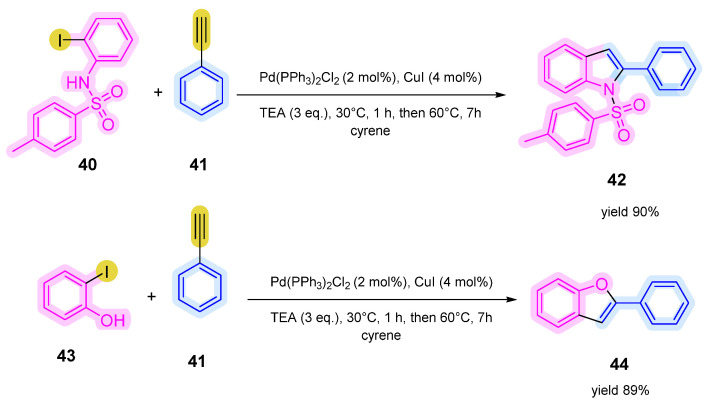
Synthesis of indole and benzofuran initiated by Sonogashira coupling in Cyrene.

**Scheme 8:**
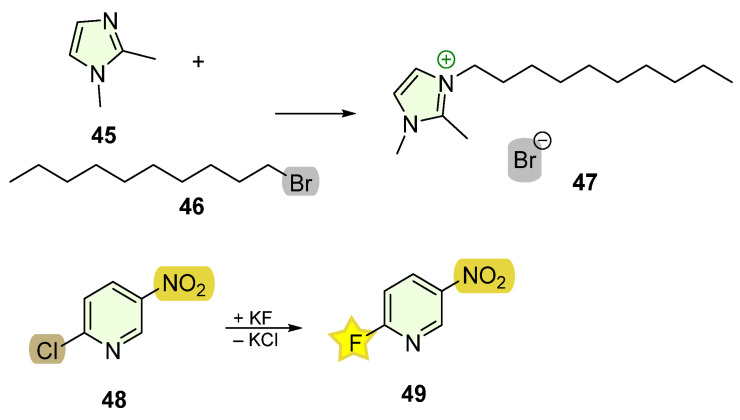
Menschutkin reaction and SN_Ar_ fluorination.

**Scheme 9:**
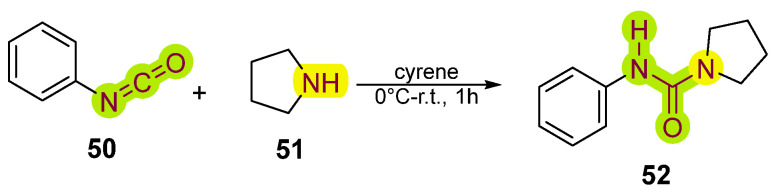
Synthesis of N,N’-substituted urea.

The versatility of Cyrene stems from the equilibrium between its ketone and geminal diol forms, which enables efficient solvation of diverse substrates through a combination of dipolar and hydrogen-bonding interactions. For instance, Cyrene has also found applications in palladium-catalyzed reactions, such as C-C bond formation via the Sonogashira and the Suzuki–Miyaura (SM) cross-coupling reactions. In particular, Cyrene proved to be a direct alternative to conventional DMF in SM coupling under mild reaction conditions involving an aryl electrophile (**37**) and an organoboron nucleophile (**38**), demonstrating optimal functional group tolerance and high yields [46]. Also, reactions between aryl iodides (**34**) and terminal alkynes (**35**) are usually reported in DMF, and Cyrene outperforms DMF in the synthesis of internal alkynes (**36**) under the same reaction conditions (Scheme 6) [47]. Sonogashira cross-coupling in Cyrene has proven its utility in the construction of bicyclic heteroaromatic frameworks, serving as a key step in the synthesis of indole (**42**) and benzofuran (**44**) derivatives (Scheme 7). Through this transformation, the desired products are obtained in excellent yields, underscoring Cyrene’s remarkable efficiency in facilitating the synthesis of heterocyclic scaffolds. Suzuki–Miyaura cross-coupling reactions have also been conducted in propylene carbonate for the synthesis of haloquinazolines in a green “environment” where the PC’s high boiling point represents an advantage, enabling heating up to 160 °C and thus shortening reaction times. Since it has low volatility, it allows the use of only small amounts of solvent [48]. Notably, Cyrene surpasses conventional solvents in nucleophilic substitution and extends its advantages even to the synthesis of ionic liquids (**47**) in the Menschutkin reaction (Scheme 8). For this class of reactions, a positive correlation exists between yield and solvent dipolarity, whereas protic solvents generally inhibit the reaction rate. Analogously, Cyrene exhibited suitable properties to promote aromatic fluorination of 2-chloro-5-nitropyridine (**48**) via SN_Ar_ reaction, although it showed the slowest kinetics among the studied solvents (Scheme 8) [25]. A convenient chromatography-free aqueous workup procedure was applied to the synthesis of N,N’-substituted ureas (**52**) derived from the addition of primary or secondary amines (**51**) to aryl isocyanates (**50**) (Scheme 9) [25].

The free miscibility with water and common organic solvents allows the application of Cyrene across a wide range of temperatures and in combination with other solvents. Due to its high viscosity, the stirring rate, co-solvent usage, and heating became relevant for the efficiency of reaction mixing. Cyrene, in the presence of a small quantity of water, was found to be a suitable medium for the bio-catalyzed esterification of benzoic acid with glycerol [49]. Despite its broad applicability, Cyrene displays notable instability in the presence of strong acids and redox-active agents. This limitation, combined with its known sensitivity to specific organic bases, significantly narrows the range of conditions under which it can be effectively employed in organic synthesis, while still representing an excellent medium for condensation or coupling reactions.

**Scheme 10:**
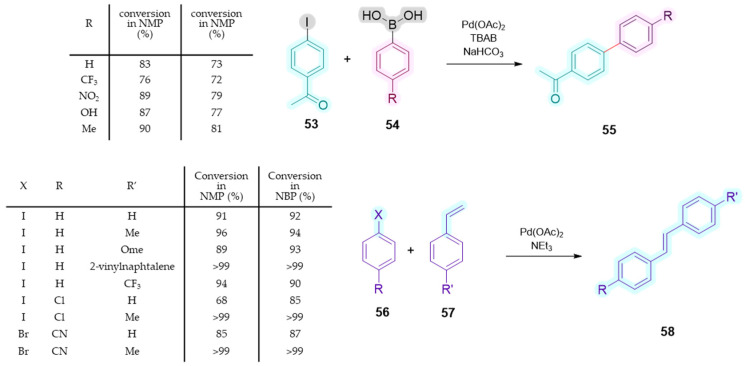
A comparison of the reaction efficiency of the Suzuki cross-coupling and the Heck reaction using NMP and NBP as solvents.

**Scheme 11:**
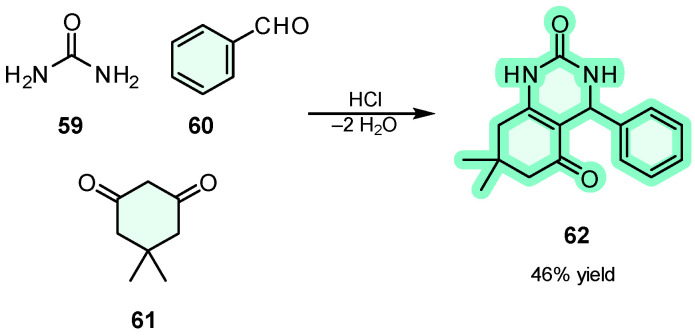
Example of Biginelli reaction in NBP.

Owing to its similarity to NMP and DMF in terms of Hansen solubility parameters and Kamlet–Taft coefficients [50], NBP was found to be a perfect “drop-in” replacement for these dipolar aprotic solvents. For instance, NBP has been explored using a range of phenylboronic acids (**54**) in Suzuki cross-coupling with 4-iodoacetophenone (**53**) and aryl halides (**56**) with terminal alkynes (**57**) in Heck cross-coupling reactions instead of its toxic analog, NMP, affording a good yield even if the reaction procedure was not optimized for this specific solvent (Scheme 10) [16]. With proper optimization of the reaction conditions, this solvent might even prove to be superior to DMF in terms of efficiency and overall performance. This solvent has also shown promising performance in both Sonogashira coupling and Biginelli reactions (Scheme 11). The yield of dihydropyrimidinone product (**62**) exceeds that obtained in conventionally employed solvents, such as ethanol and DMF [16]. Its application in solution-phase synthesis is somewhat limited by its high boiling point (241 °C), as product isolation requires well-designed workup procedures to crystallize and isolate the product by filtration. This constraint, however, turns into an advantage for solid-phase applications, where a filtration-based procedure aligns with greener and safer laboratory practices.

### 4.2. Solid-Phase Peptide Synthesis (SPPS)

**Figure 8:**
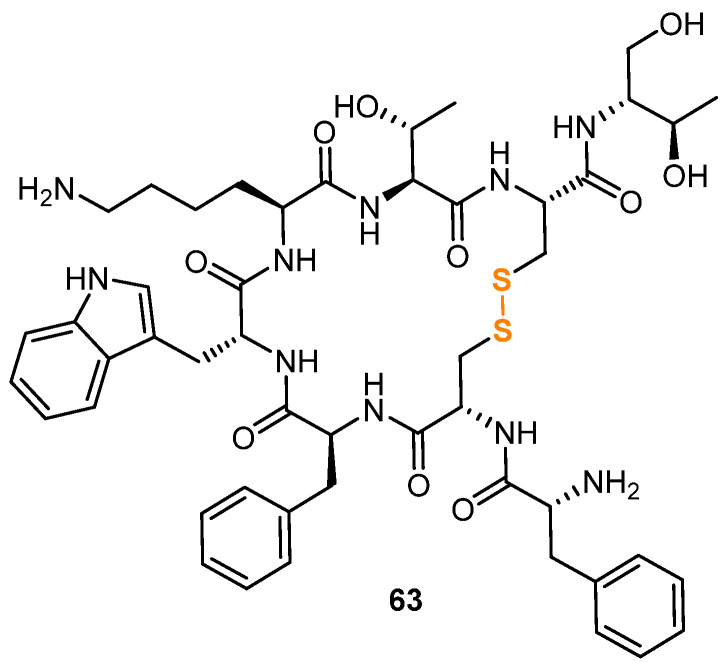
Structure of Octreotide (**63**).

In SPPS, the use of NBP has demonstrated significant advantages. Albericio and co-workers reported that NBP can replace DMF in Fmoc-based SPPS, yielding peptides of comparable or higher purity, while reducing hazards. Likewise, DMSO has been employed in peptide elongation steps, showing improved coupling efficiency with sterically hindered amino acids and reduced racemization [51]. Although DMSO has been shown to have a low swelling capacity, it reduces reaction rates and resin accessibility. For this reason, in peptide synthesis, it is primarily employed as a primary solvent or in a mixture, to run specific reactions such as oxidation, cyclization, or 1,3-dipolar addition known as CuAAC click reactions. On the other hand, NBP is compatible with the most popular polystyrene-based resins, classical supports used in the large-scale manufacture of peptides. The use of this solvent affords peptide crudes of comparable quality to those obtained in DMF, as demonstrated by a feasibility study on the synthesis of Octreotide (**63**) performed in NBP (Figure 8) [17].

In stepwise SPPS, the impurities detected when using NBP were mainly deletion sequences, likely arising from the slower coupling kinetics of activated amino acids in this solvent. One of the major challenges is reduced coupling efficiency with Fmoc-Arg(Pbf)-OH, which is likely due to intramolecular lactamization of arginine during its activation in NBP. This side reaction is also known to occur in DMF; however, because couplings proceed significantly faster in DMF, complete incorporation of arginine can typically be achieved with a single or, at most, a double coupling. In contrast, the slower kinetics observed in NBP often require multiple coupling cycles, necessitating double or even triple couplings to achieve complete acylation. The reduced coupling rates may also be attributed to the difference in viscosity between the two solvents. NBP is considerably more viscous than DMF (4.0 vs. 0.8 cP at 25 °C). In small, automated peptide synthesizers, this higher viscosity slows solvent transfers and requires adjusted cycle times to ensure complete fluid handling. By contrast, with large-scale equipment, this feature is far less problematic. To improve NBP’s performance in SPPS, carrying out the synthesis at elevated temperature has emerged as a promising approach. Indeed, pre-conditioning the resin with 25% of the total NBP at 45 °C allows adequate swelling before coupling. This precaution facilitates the formation of the Oxyma ester when the resin is fully solvated, thereby reducing the likelihood of concurrent δ-lactam cyclisation. The protocol was successfully applied to the synthesis of a cyclic RGD peptide (H-DfKRG-OH), a precursor of Cilengitide [18].

**Figure 9:**
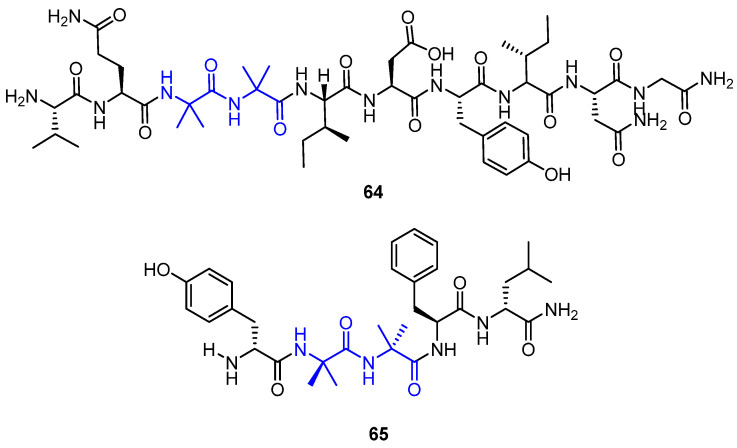
Structures of Aib-ACP decapeptide (**64**) and Aib-enkephalin pentapeptide (**65**).

The replacement of DMF with γ-valerolactone (GVL) in SPPS has been demonstrated by Albericio and coworkers, who manually synthesized two demanding model peptides using a PS-based resin: the Aib-ACP decapeptide (**64**) (H-Val-Gln-Aib-Aib-Ile-Asp-Tyr-Ile-Asn-Gly-NH_2_) and the Aib-enkephalin pentapeptide (**65**) (H-Tyr-Aib-Aib-Phe-Leu-NH_2_) (Figure 9).

**Figure 10:**
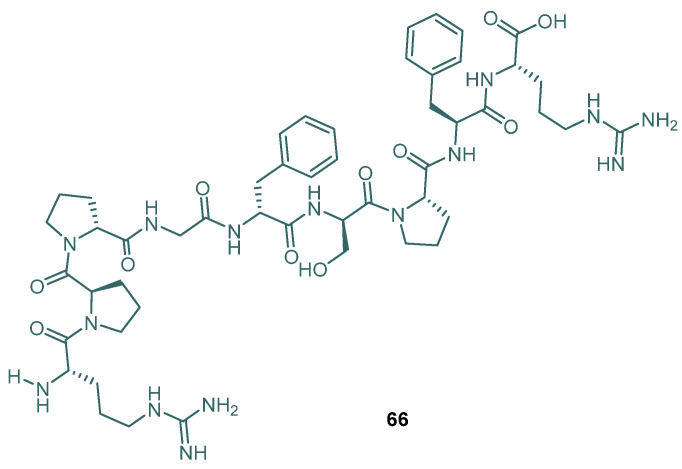
Structures of Bradykinin (**66**).

In the developed protocols, GVL has successfully substituted DMF in coupling conditions using DIC/Oxyma strategies, with no detection of major side-reactions associated with ring opening and subsequent acylation of the amino functions [52]. Also, Propylene carbonate (PC) has been explored as another promising green alternative in SPPS. Its physicochemical properties enable both solution- and solid-phase peptide synthesis, supporting the use of acid- or base-labile protecting groups. Importantly, activated amino acids exhibit minimal racemization in this medium, highlighting its chemical compatibility with standard peptide-coupling conditions. The feasibility of SPPS in PC has been validated through the successful assembly of the nonapeptide Bradykinin (**66**) (H-Arg-Pro-Pro-Gly-Phe-Ser-Pro-Phe-Arg-OH), demonstrating its potential as a sustainable alternative to DMF and related solvents (Figure 10) [53].

Building on this, more recently, the suitability of propylene carbonate has also been confirmed in continuous-flow solid-phase peptide synthesis (CF-SPPS), enabling a rapid, scalable, and markedly more sustainable process. This technology was first tested for the synthesis of various α-peptides and then examined with more challenging sequences, including β-peptide foldamers and α/β-mixed backbones. What makes this study particularly fascinating is the demonstrated scalability up to the 4 g range, which significantly enhances the industrial appeal of this technology [54].

### 4.3. Microwave-Assisted Synthesis

Microwave-assisted protocols have been successfully coupled with green solvents to enhance reaction efficiency in both peptide synthesis and conventional organic transformations. Among them, Cyrene has demonstrated compatibility with microwave-assisted N-arylation and C–C coupling reactions, achieving efficiency comparable to that of DMF while reducing environmental impact [55].

**Scheme 12:**
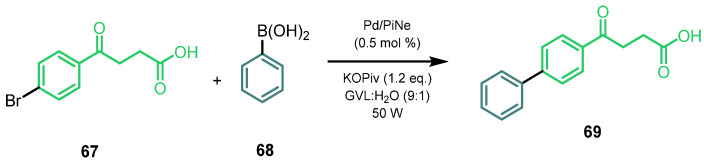
Optimized synthesis of the Fenbrufen API.

Cyrene, as a sustainable reaction medium, finds application in heterocyclic multicomponent chemistry. Starting from chromone derivatives, aliphatic or aromatic amines, and phenyl- or pyridylacetonitriles, a wide variety of bipyridine analogs could be assembled in a one-pot reaction. Notably, conducting the process in Cyrene at elevated temperatures (150 °C) under microwave irradiation enabled a catalyst- and additive-free protocol, significantly accelerating the transformation and delivering the desired products in good to excellent yields [56]. Microwave irradiation not only increases reaction yield but also addresses solvent-related issues, such as viscosity and reaction mixture homogeneity. In this regard, GVL has been considered as a promising blending solvent, with some use also in reducing the viscosity of dihydrolevoglucosenone [57]. Moreover, GVL has proven to be a suitable medium for microwave-driven amidation and esterification, enabling significantly shorter reaction times and consistently high yields. In the area of heterogeneous metal-based catalysts, GVL has been exploited in a Suzuki–Miyaura cross-coupling process with a circular economy approach using a biowaste-derived heterogeneous catalyst, Pd/PiNe. Microwave irradiation significantly enhanced energy efficiency, enabling the synthesis of various biphenyls and reducing reaction time. The optimized procedure well tolerates the free carboxylic acid group; thus, it has been applied to achieve a “step-economical preparation” in a quantitative yield of the non-steroidal anti-inflammatory analgesic Fenbrufen (**69**) (Scheme 12) [39].

Microwave-assisted SPPS has shown that higher temperatures accelerate coupling reactions and enhance overall efficiency, even for intricate peptides. NBP emerged in several studies as a robust candidate to replace DMF in microwave-assisted SPPS, demonstrating reliable performance even with structurally demanding peptides, to the point that some manufacturers of automated synthesizers now list it among their recommended green solvents [58]. Nonetheless, NBP is not the sole solvent capable of completely substituting DMF. GVL has also been successfully implemented in automated microwave-assisted SPPS, following protocols that offer notable improvements in energy efficiency, safety, and solvent economy.

In this view, the successful synthesis of structurally demanding peptides, such as the Jung–Raderman (JR) decapeptide (H-WFTTLISTIM-NH_2_), the ABC peptide (H-VYWTSPFMKLIHEQCNRADG-NH_2_), and Thymosin β-derived sequences (e.g., H-SDAAVDTSSEITTKDLKEKKEVVEEAEN-NH_2_), highlights the robustness of GVL under microwave irradiation using both polystyrene- and PEG-based solid support [59]. The major limitation of alternative solvents lies in their physicochemical properties, particularly viscosity and dielectric behavior, which directly influence both resin swelling and microwave heating efficiency. To overcome these intrinsic limitations, an emerging, highly effective strategy is the use of binary solvent mixtures.

### 4.4. Ultrasound-Assisted Synthesis

**Figure 11:**
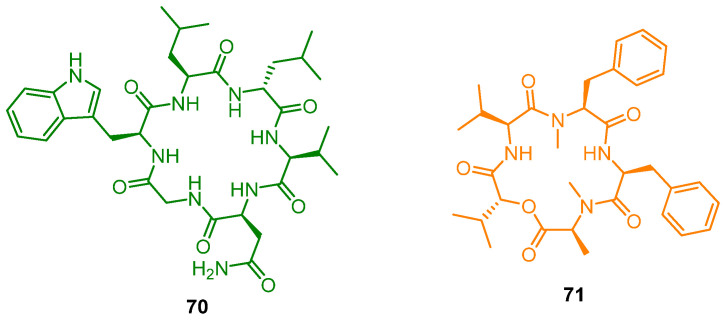
Natural products synthesized via US-GSPPS.

Ultrasound has been increasingly explored with alternative solvents, enhancing mass transfer and reaction rates. For example, GVL has been applied as a medium for ultrasound-assisted esterification and transesterification, demonstrating improved reaction kinetics and reduced catalyst loading [60]. Similarly, DMSO has been tested in sonochemical oxidation reactions, providing higher yields and selectivity compared to conventional solvents [61]. Ultrasonication has been investigated for the synthesis of challenging peptides and natural products via on-resin esterification and solution- and solid-phase macrocyclization using a versatile N-formylmorpholine (NFM)/Anisole (An) 1:1 solvent mixture. The total synthesis of Desotamide B (**70**) and Acremonamide (**71**) via US-GSPPS paves the way for advancing green chemistry practices and industrial applications in peptide synthesis (Figure 11) [62].

### 4.5. Green Binary Solvent Mixture

Sometimes, a “drop-in” solvent replacement may be sufficient to migrate away from hazardous solvents; other times, a solvent mixture may be the best choice to meet the reactions’ requirements. In SPPS, the Fmoc cleavage reaction is effective in polar aprotic solvents, like DMF, NMP, and DMSO, while displaying a lower reaction rate in apolar aprotic solvents, like THF, anisole, and EtOAc. On the other hand, the coupling reaction requires apolar, aprotic solvents to be efficient, and resin swelling should not be overlooked, making it difficult to find a single neat solvent suitable for all steps in SPPS. In this view, carefully designed combinations of bio-based solvents and greener organic co-solvents can balance dielectric properties, improve mass transfer, and increase resin accessibility, thus overcoming many of the constraints observed with single-solvent systems. For example, despite its favorable sustainability profile, the use of Cyrene in SPPS remains limited due to several intrinsic drawbacks, including its high viscosity, which limits resin swelling and mass transfer. Mixtures of Cyrene/diethyl carbonate (30:70), sulfolane/diethyl carbonate (30:70), and anisole/dimethyl carbonate (70:30) exhibit excellent swelling capacity for both PS- and PEG-based resins, independent of functionalization. These mixtures also dissolve a broad range of amino acids efficiently and perform exceptionally well when paired with DIC/Oxyma Pure as the coupling system. The practical utility of this approach was demonstrated through the synthesis of demanding peptides. For instance, anisole/dimethyl carbonate (70:30) enabled the preparation of Aib-enkephalin with satisfactory HPLC purity on ChemMatrix-Rink Amide resin. It also supported the successful assembly of both Aib-ACP and linear Octreotide, representing a valid alternative to DMF [63].

These syntheses have also been successfully performed using a mixture of GVL and N-formylmorpholine (NFM) in polystyrene-based SPPS, although careful examination of the solvating properties of the reagents was required [52].

Varying the composition of the green binary solvent mixture can be employed to mitigate common side reactions, such as Arg-lactamization, amino acid epimerization, and aspartimide formation. As NBP requires attention to avoid lactamization of the arginine residue, efforts were made to determine whether this side reaction could be avoided by tuning solvent conditions during SPPS. Increasing the solvent polarity with binary mixtures composed of DMSO/2-Me-THF (tetrahydro-2-methylfuran) (4:6) and DMSO/DOL (1,3-dioxolane) significantly reduces the formation of δ-lactam species. The latter, at different solvent ratios, also suppresses aspartimide formation. Generally, binary solvent mixtures of DMSO with DOL, 2-Me-THF, or EtOAc are suitable for efficient Fmoc removal. In contrast, coupling reactions can be envisioned in these mixtures as well as in NFM/DOL and NBP/DOL [64]. NBP associated with EtOAc or 2-Me-THF demonstrated performance on par with DMF in the synthesis of [Asp^5^]-Vasopressin [58].

Such mixtures can enhance reaction rates, improve selectivity, and mitigate side reactions, extending their utility well beyond peptide chemistry to include cross-coupling reactions, condensations, and other transformation classes. Green solvents have been expertly combined in organic synthesis, for example, adding water as a cosolvent to Cyrene appears to increase the fluidity of the reaction mixture, reducing stirring issues and preventing solidification. This system provides a valid medium for Suzuki–Miyaura cross-coupling at 50 °C [46]. In addition, the widespread use of aqueous–organic solvent systems for Suzuki reactions stems from water’s ability to solvate the halide byproduct, thereby reducing its interference with the reaction’s progress. In this respect, great efforts were made to replace organic solvents and mixtures with in-water amide formation, even applied to organic and peptide chemistry [65]. Although these methodologies offer clear operational advantages, they can also lead to increased production costs and substantial challenges during waste management. The disposal of large quantities of aquatic waste containing organic and inorganic substances requires procedures that are far from ecological, adding complications to this framework that must be considered. Overall, the generation of mixed solvent waste complicates and increases the cost of recycling; thus, a total replacement with a single solvent remains the best choice from an environmental perspective, especially for SPPS, given its substantial solvent demand.

### 4.6. Practical and Economic Considerations: Downstream Processing and Life Cycle Perspective

While chemical efficiency and reaction performance are critical parameters, the practical adoption of alternative solvents to DMF also depends strongly on economic and downstream processing considerations. Several of the solvents discussed in this review exhibit significantly higher boiling points than DMF, which can complicate solvent removal and product isolation. In laboratory-scale protocols, this often necessitates energy-intensive evaporation under reduced pressure or multi-step aqueous workup procedures, potentially offsetting some of the environmental benefits associated with reduced toxicity. In contrast, DMF—despite its regulatory concerns—remains relatively easy to remove and recycle under established industrial conditions. These downstream limitations of high-boiling polar solvents have been widely discussed in the context of green solvent substitution, particularly for DMSO and GVL, where solvent recovery and purification represent non-negligible energetic and operational burdens [66].

From an economic perspective, the cost competitiveness of bio-based solvents is highly dependent on production scale and feedstock availability. Solvents such as GVL, Cyrene^®^, and propylene carbonate benefit from renewable or waste-derived feedstocks, but their current market prices may exceed those of traditional dipolar aprotic solvents. However, increasing industrial demand and process optimization are expected to progressively reduce costs, as already observed for several bio-based platform chemicals. Techno-economic analyses indicate that large-scale deployment and process intensification are key drivers for cost reduction in bio-based solvents, particularly for GVL and Cyrene^®^, whose production routes are still evolving [67].

Importantly, the sustainability of solvent substitution cannot be assessed solely on toxicity or renewability criteria. Life cycle assessment (LCA) studies have shown that the overall environmental impact of a solvent is determined by a combination of factors, including energy consumption during production, recyclability, solvent losses, and end-of-life fate. In this regard, bio-based solvents do not automatically guarantee a lower environmental footprint compared to DMF, and careful case-by-case evaluation remains essential. These considerations underline the importance of integrating solvent selection with downstream processing strategies and solvent recovery protocols to achieve genuinely sustainable synthetic processes. Recent LCA-driven solvent selection frameworks emphasize that high boiling points, energy-intensive recovery, or limited recyclability can significantly impact the overall environmental balance, even for solvents derived from renewable resources [68,69].

## 5. Conclusions and Future Directions

The growing awareness of the health, environmental, and regulatory challenges associated with long-standing solvents such as DMF, NMP, and other dipolar aprotic media has made the search for safer replacements a compelling priority. Evidence accumulated over the last decade clearly shows that many traditional solvents present risks that are no longer compatible with modern sustainability standards, especially in fields such as peptide synthesis, where solvent volumes are exceptionally high. Consequently, the use of greener, less hazardous alternatives is no longer a theoretical exercise but a concrete and achievable goal.

In this review, we have outlined the most promising bio-based and lower-toxicity solvents, examining their physicochemical properties, compatibility with key synthetic transformations, and practical advantages and limitations. Their feasibility in conventional reactions used in organic and peptide synthesis has been discussed to highlight that selecting more sustainable options is possible without compromising reaction efficiency or product quality. Greener solvents, whether employed individually or as carefully designed mixtures, are increasingly able to match, and in some instances surpass, the performance of traditional systems.

Beyond DMF, NMP, and 1,4-dioxane, other compounds such as trifluoroacetic acid (TFA) are also being scrutinized due to emerging environmental and safety concerns. The progress made in identifying viable replacements strongly suggests that the scientific community will be well positioned to adapt to future restrictions, including the possible need to reduce or replace TFA in peptide workflows.

It is worth noting that water, as the most abundant and environmentally benign solvent available, represents an ideal benchmark for sustainability in chemical synthesis. Significant efforts have been devoted to developing organic reactions in aqueous media, and water-based methodologies have demonstrated remarkable potential in selected transformations. In this context, recent studies on chemical reactivity in water microdroplets have attracted considerable attention, revealing unusual rate accelerations and reactivity enhancements at air–water interfaces [70,71,72,73]. These findings highlight the conceptual potential of aqueous systems for sustainable synthesis.

However, despite these advances, the widespread replacement of DMF with water in peptide synthesis remains highly challenging. To date, no generally applicable and robust peptide coupling strategies in purely aqueous media are available, as most commonly used activating agents and coupling reagents display limited stability or efficiency in the presence of water.

An alternative promising direction involves the development of solution-phase peptide synthesis strategies that rely on soluble supports or molecular tags, enabling more efficient purification, reduced solvent usage, and smoother integration with continuous-flow technologies [74,75,76]. Such approaches may offer a powerful means of reducing reliance on traditional solvents while maintaining—or even improving—overall process efficiency. The transition from DMF to alternative solvents presents both challenges and opportunities. While many proposed candidates offer similar or superior solubilizing properties, their compatibility with existing synthetic methodologies and their scalability in industrial applications require further investigation. Moreover, the environmental impact of producing and disposing of these alternative solvents must be carefully evaluated to ensure that they provide a true net benefit over DMF within a life-cycle perspective.

Overall, the search for sustainable alternatives to DMF is crucial for advancing green chemistry principles in peptide and organic synthesis. Several promising candidates have already been identified, but further research is needed to fully understand their properties, applications, and long-term environmental impacts. Close collaboration between academia and industry will be essential to facilitate the adoption of these alternative solvents in practical settings. Future research should focus on:

- Developing new solvents that combine low toxicity, high solubilizing power, and environmental sustainability;
- Investigating the scalability of alternative solvents in industrial applications;
- Assessing the life-cycle environmental impact of alternative solvents;
- Exploring the compatibility of alternative solvents with a broader range of synthetic methodologies.

Taken together, these advances indicate that replacing traditional dipolar aprotic solvents is not only feasible but also strategically necessary to ensure that peptide and organic synthesis evolve toward safer, cleaner, and fully sustainable processes.

## Data Availability

No new data were created or analyzed in this study. Data sharing is not applicable to this article.
